# The knowledge paradox: an inverted U-shaped association between HIV knowledge and stigma among older men in Sichuan Province, Southwest China

**DOI:** 10.3389/fpubh.2025.1685602

**Published:** 2025-12-03

**Authors:** Zhihua Ye, Jin Peng, Shu Liang, Yuan Li, Jiang Yuan, Ruixi Zhang, Jia He, Shuangru Li, Bihui Yang, Xiaochun Zhang, Yi Yang

**Affiliations:** 1 School of Management, Chengdu University of Traditional Chinese Medicine, Chengdu, Sichuan, China; 2 Institute for the Prevention and Control of Sexually Transmitted Diseases and AIDS, Sichuan Center for Disease Control and Prevention, Chengdu, Sichuan, China; 3 School of Public Health, Chengdu University of Traditional Chinese Medicine, Chengdu, Sichuan, China

**Keywords:** HIV knowledge, stigma, inverted U-shaped association, older men, sociocultural factors

## Abstract

**Background:**

Older men (≥50 years) in China face elevated HIV infection risks, yet HIV stigma remains a significant barrier to prevention. Although HIV knowledge is frequently assumed to reduce stigma, the evidence is inconsistent. This cross-sectional study examined the association between HIV knowledge and stigma among older men in Southwest China, specifically investigating potential nonlinear patterns within sociocultural contexts.

**Methods:**

We conducted a cross-sectional survey from July to August 2023 in three high-HIV-burden areas of Sichuan Province. Using a multi-stage cluster random sampling design, 841 HIV-negative men completed structured interviews. All analyses incorporated survey weights to ensure representativeness. HIV knowledge was assessed via a validated 8-item scale (score range 0–8). Stigma was measured with an adapted 10-item scale (score range 10–50). Survey-weighted hierarchical linear regression was used to test linear and quadratic associations adjusted for covariates. Survey-weighted segmented regression analyses confirmed robustness.

**Results:**

The weighted mean HIV knowledge score was 4.39 (SD = 2.13), and the weighted mean stigma score was 31.78 (SD = 5.60). Regression analysis revealed an inverted U-shaped association. Stigma initially increased with increasing knowledge (linear *β* = 1.71, *p* < 0.001), peaked at a knowledge score of 4.14, and subsequently decreased with increasing knowledge gain (quadratic *β* = −0.21, *p* < 0.001). Robustness analyses confirmed differential knowledge-stigma associations across knowledge levels (interaction *β* = −1.47, *p* < 0.001). Lower stigma was independently associated with non-rural household registration (*β* = −2.39, p < 0.001), access to a greater number of HIV health education channels (2–3 types: *β* = −1.07, *p* < 0.01; ≥4 types: *β* = −2.29, *p* < 0.05), and more liberal sexual attitudes (*β* = −0.19, *p* < 0.001). Social support and socioeconomic status were not associated.

**Conclusion:**

Among older Chinese men, HIV knowledge has an inverted U-shaped association with stigma. Initial knowledge acquisition correlates with heightened stigma, potentially reflecting sociocultural interpretations of fragmented information. Only beyond a specific threshold does further knowledge correlate with reduced stigma. Interventions may need to be staged or tailored based on individuals’ existing knowledge levels, addressing both the nonlinear knowledge-stigma dynamic and underlying sociocultural norms.

## 1 Introduction

Globally, the proportion of people living with HIV (PLWH) aged ≥50 years has risen significantly, driven by expanded antiretroviral therapy (ART) access and increasing new infections among older adults (1, 2). Similarly, China reflects this trend, with a sustained increase in older PLWH, particularly men (3, 4). Multiple factors contribute to this pattern. Unlike women, men often remain sexually active beyond age 50, yet persistent sexual needs in this group are frequently overlooked due to traditional sociocultural norms in China (4, 5). Thus, limited education, inadequate HIV knowledge, low-risk awareness, and infrequent condom use increase the likelihood of engaging in unprotected high-risk sex (4). Such behaviors not only increase individual infection risk but also facilitate HIV transmission to sexual partners, positioning older men as a potential “bridge population” between high-risk groups (e.g., female sex workers) and lower-risk partners (e.g., spouses) (3). Furthermore, studies in this context indicate that the sexual networks of this demographic include both peer and middle-aged female sex workers, a pattern that may amplify HIV transmission dynamics (3, 6). Recognizing these vulnerabilities, China designated older adults as a priority group for national HIV/AIDS prevention in 2017 (6). Despite this focus, late HIV diagnosis rates among older PLWH remain substantially higher than those in younger populations (7), impeding progress toward 95–95–95 targets and underscoring the need to identify barriers to prevention.

HIV stigma is a well-documented barrier to achieving 95–95–95 targets (8). Consistent evidence links stigma to delayed HIV testing, reduced use of prevention services, lower ART adherence, heightened psychological distress, and poorer quality of life among PLWH (9–11). Stigma manifests primarily as public stigma (negative attitudes toward and discrimination against PLWH in the general population) and self-stigma (internalization of societal prejudice by PLWH) (12–14). The evidence suggests that self-stigma originates from socially transmitted public stigma (15, 16), indicating that reducing public stigma may be pivotal for mitigating the overall impact of stigma. Despite four decades of HIV/AIDS advocacy, public stigma persists globally — recent 2024 data show a median prevalence of stigmatizing attitudes toward PLWH of 53%, far exceeding the UNAIDS (Joint United Nations Programme on HIV/AIDS) target of <10% (17). This pervasive public stigma may obstruct testing and treatment access while exacerbating self-stigma, perpetuating cycles of HIV transmission (18).

While numerous studies have examined drivers of HIV public stigma, findings on key determinants remain inconsistent. Many authors attribute stigma primarily to gaps in HIV knowledge, which are often influenced by sociodemographic factors, and report a negative association between knowledge and stigmatizing attitudes (19–22). However, the strength and direction of this relationship vary across populations and contexts. Some studies note persistent stigma even among highly knowledgeable individuals, showing weak or non-significant negative associations (23–25). This implies that deeper sociocultural norms, socioeconomic inequalities, and power dynamics may shape stigma formation (18). Intriguingly, research in conflict-affected settings revealed a paradoxical positive association between knowledge and stigma at lower knowledge levels, contrasting with the expected negative association at higher levels (26). These inconsistencies highlight methodological limitations — studies focusing narrowly on knowledge gaps may overlook contextual complexity, whereas those integrating sociocultural factors often rely on linear models that inadequately capture dynamic relationships.

Among older Chinese men, a group exhibiting high HIV stigma toward PLWH (27, 28), unique sociocultural vulnerabilities exist: limited education and restricted HIV information access reduce campaign effectiveness (29); adherence to traditional sexual taboos impedes open discussion (5, 28); and fear-based HIV/AIDS messaging from the late 20th century may perpetuate internalized stigma (30). These intersecting factors necessitate examining how sociocultural context influences stigma in this population. Given the potential limitations of assuming linearity, analyses should explicitly explore nonlinear dynamics between HIV knowledge and stigma.

Therefore, this cross-sectional study aimed to do the following:

1. Examine the association between HIV knowledge and stigma among older men in Sichuan Province, Southwest China, within its sociocultural context.
2. Test for a potential nonlinear relationship between HIV knowledge and stigma.

These findings may inform culturally appropriate antistigma interventions for this population.

## 2 Method

### 2.1 Study design and setting

We conducted a cross-sectional study in Sichuan Province, Southwest China, from July to August 2023 to examine HIV knowledge and stigma among men aged ≥50 years. Sichuan has the highest burden of PLWH in China, with over 60% of PLWH in some cities being older adults (4, 31). Collaborating with the Sichuan Provincial Center for Disease Control and Prevention (CDC), we selected three geographically and socioeconomically diverse areas identified by the National HIV/AIDS Comprehensive Response Information Management System as having high concentrations of older PLWH: Chengdu, Yibin, and Liangshan.

Recruitment and data collection co-occurred from July 21 to August 25, 2023, across primary healthcare institutions (community health centers and township hospitals) in all three urban and rural areas. Trained interviewers administered questionnaires onsite.

A pilot survey was conducted on July 11, 2023, in a Chengdu community health center to refine instruments and procedures and train interviewers. Pilot data were excluded from the formal analysis. Following this preparation, the formal survey was expanded to all the sites in Chengdu, Yibin, and Liangshan.

### 2.2 Participants

We recruited participants from Chengdu, Yibin, and Liangshan via a three-stage cluster random sampling approach. First, we randomly selected three townships, towns, or subdistricts from Chengdu’s County A, Yibin’s District B, and Liangshan’s County C, respectively. Second, one village or community was randomly chosen within each selected township, town, or subdistrict. Finally, one villager or resident group was randomly selected per village or community. The first two stages of selection were based on official administrative rosters, while the final stage utilized the resident rosters available to and maintained by local government staff. Government staff invited all potentially eligible men in these groups to participate at local primary healthcare institutions.

Eligibility required participants to (1) be aged ≥50 years, (2) identify as male, (3) be local residents, and (4) have confirmed HIV-negative status (per standardized testing protocols in the HIV Testing section). The exclusion criteria included cognitive impairment, severe illness, or communication barriers. Only participants who reported having heard of HIV/AIDS were included in the final analysis.

After providing written informed consent, eligible participants completed private, 30-min interviews conducted by interviewers who were locally fluent in the language. All the interviewers underwent standardized training to ensure consistent interview administration. The questionnaires were reviewed immediately for completeness. The participants received 15 CNY (approximately 2 USD) of compensation, regardless of whether they completed the interview.

### 2.3 HIV testing

The HIV testing adhered to China’s National Guideline for the Detection of HIV/AIDS. Primary healthcare institutions conducted initial screening via two parallel approaches: onsite rapid testing at designated points via a colloidal gold rapid test (Dot Immunocolloid Gold Rapid Test, InTec PRODUCTS, INC) or venous blood collection for HIV antibody detection via enzyme-linked immunosorbent assay (ELISA) at local screening laboratories. A reactive result from either initial screen required collecting a new venous blood sample. These samples were then sent to designated confirmation laboratories for definitive protein immunoblotting testing (western blot).

### 2.4 Variables and measurement

The questionnaire incorporated both published and self-developed instruments. To ensure content validity and cultural relevance, we consulted experts in sociology and epidemiology during the development of the instrument. Pilot and formal survey testing confirmed the acceptable reliability of all the measures, with internal consistency results detailed later. English versions of all instruments are available in Supplementary Material 1.

#### 2.4.1 Independent variable

The HIV knowledge was assessed via the validated HIV Knowledge Scale from the Chinese CDC, a tool widely implemented within China’s national HIV/AIDS surveillance system (5, 27). This 8-item scale is awarded 1 point for each correct answer and 0 points for incorrect or “do not know” responses, yielding a total score where higher values reflect greater knowledge. The scale demonstrated good reliability (Cronbach’s alpha = 0.79).

#### 2.4.2 Dependent variable

The HIV stigma was measured via a validated Chinese adaptation of Zelaya’s HIV Stigma Scale (32, 33). While the original 24-item scale covers four domains (fear of infection, shame and prejudice, personal stigma, and social stigma), pilot testing indicated that some items were culturally irrelevant for older Chinese men, leading to a refined 10-item version. The responses used a five-point Likert scale, with higher total scores indicating stronger stigma toward PLWH. The reliability was also good (Cronbach’s alpha = 0.77).

#### 2.4.3 Covariates

The covariates were categorized into three groups (detailed classifications in Table 1).

**Table 1:** Sociodemographic and HIV-related characteristics of the participants (unweighted *n* = 841).

Variable	*n* (%)
City	
Chengdu	407 (30.62)
Yibin	357 (63.40)
Liangshan	77 (5.98)
Age (years)	
50–59	379 (41.16)
60–69	262 (31.41)
≥70	200 (27.42)
Ethnic groups	
Han	816 (98.19)
Ethnic minority	25 (1.81)
Household registration	
Rural	740 (90.57)
Non-rural	101 (9.43)
Education	
No education	107 (12.22)
Primary school	363 (45.26)
Junior high school	283 (32.84)
High school and above	88 (9.68)
HIV/AIDS health education (channel types)	
0–1	432 (57.23)
2–3	313 (34.01)
≥4	96 (8.75)
Monthly income (CNY)	
No income	92 (11.38)
<1,000	301 (46.40)
1,000–1999	167 (15.71)
2000–2,999	126 (11.77)
3,000–3,999	87 (8.25)
≥4,000	68 (6.50)
Occupation	
Unemployed or out of work	100 (9.38)
Basic production	648 (81.02)
Service occupations	54 (5.00)
Professional, technical, or managerial	39 (4.60)
Marital status	
Married	695 (82.79)
Unmarried	56 (5.68)
Divorced or widowed	90 (11.53)
Residential status	
Alone	147 (17.98)
With spouse or family only	437 (56.07)
With spouse and family	257 (25.95)
Self-reported HIV testing	
Not tested	775 (94.53)
Tested	66 (5.47)

n = unweighted count; % = weighted percentage. All percentages were calculated using survey weights to represent the target population.

Sociodemographic variables included city of residence, age, ethnicity, household registration type, education level, monthly income (incorporating financial support from children), occupation, marital status, and living situation.

HIV-related characteristics included the number of different HIV health education channels accessed and self-reported history of HIV testing. The assessment of health education channels encompassed a broad range of sources, including institutional (e.g., the CDC, hospitals), media-based (e.g., television and the internet), and interpersonal channels (e.g., family and volunteers).

Sociocultural variables included sexual attitudes, social support, and socioeconomic status (SES). Sexual attitudes, measured via a four-item original scale, assess views on non-marital sexual behavior via a five-point Likert scale; higher scores indicate more liberal attitudes (Cronbach’s alpha = 0.86). Social support, assessed via a two-item original instrument, measures perceived economic and emotional support from family, friends, or social networks on a five-point Likert scale; higher scores denote stronger perceived support (Cronbach’s alpha = 0.85). SES was calculated as a composite score based on education level, income, and occupation (using preretirement occupation for retired participants) (34, 35). These three indicators were standardized (*Z* scores). Principal component analysis yielded one component (eigenvalue = 1.710) with factor loadings of 0.725 (education), 0.780 (income), and 0.760 (occupation). The SES score was derived as follows: [(0.725 * Z_education + 0.780 * Z_income + 0.760 * Z_occupation)/1.710], with higher scores indicating a higher SES.

### 2.5 Statistical methods

#### 2.5.1 Survey design and weighting

To ensure the representativeness of our findings and address the complex multistage cluster sampling design, we performed all analyses using survey weighting methods. The design was treated as a two-stage stratified cluster sample, with cities (Chengdu, Yibin, and Liangshan) as strata. Within each city, townships, towns, or subdistricts served as primary sampling units (PSUs), and villages or communities within these PSUs were selected as secondary sampling units (SSUs). The original three-stage design was simplified to two stages for analysis, as the primary clustering and variation were captured within these two stages. Additionally, subsequent villager or resident groups were not formal administrative units, preventing accurate specification of a third-stage sampling frame and population size. Sampling weights were applied using the svyset command in Stata, incorporating selection probabilities and finite population corrections at each stage.

#### 2.5.2 Data management

All the analytical variables were checked for missing data, and no missing data were present in the final sample (*n* = 841). To ensure accuracy, all the data were subjected to double entry and validation via EpiData software (version 3.1) before analysis.

#### 2.5.3 Descriptive and bivariate analyses

All descriptive and bivariate analyses accounted for the complex survey design. Categorical variables were summarized as unweighted counts (n) and weighted percentages (%). Continuous variables were presented as weighted means with their corresponding weighted standard deviations (SD). This approach aligns with standard practices for complex survey data. Bivariate associations with HIV stigma were assessed using survey-weighted linear regression. For continuous independent variables, the analysis provided unstandardized coefficients (*β*) and their standard errors (SE). For categorical independent variables, group differences were evaluated with an overall survey-weighted *F*-test. When a significant association was identified, post-hoc pairwise comparisons were conducted using adjusted Wald tests.

#### 2.5.4 Primary analysis

The association between HIV knowledge and stigma was examined using hierarchical linear regression, with all models incorporating the survey weights. Variance estimation was accounted for using Taylor linearization, the default method in Stata, in the complex survey design. Model 1 was adjusted for sociodemographic factors (Table 1), HIV-related characteristics (health education channels accessed, HIV testing history), and sociocultural variables (social support, sexual attitudes, SES). Covariates were retained in all models to control for potential confounding, irrespective of statistical significance (36). Model 2 added HIV knowledge as a linear term. Model 3 introduced a quadratic term (HIV knowledge × HIV knowledge) to evaluate nonlinearity formally. Given the design constraints, we focused on the significance of the regression coefficients for interpretation. The turning point of the curve was derived by setting the first derivative of the quadratic equation to zero, with significance confirmed via Stata’s utest command (37). All the models report unstandardized *β* coefficients with standard errors (SE).

#### 2.5.5 Robustness checks

Survey-weighted segmented regression and interaction analyses were performed to validate the stability of the observed inverted U-shaped relationship. The participants were stratified into lower-knowledge (score ≤ 4.14) and higher-knowledge (score > 4.14) groups based on the turning point identified by the utest command (knowledge score = 4.14). Linear associations between knowledge and stigma were then examined separately within each subgroup using survey-weighted linear regression. To formally test whether the association between knowledge and stigma differed significantly across these groups, an interaction term (HIV knowledge group × HIV knowledge) was added to a survey-weighted linear regression model using the full sample.

### 2.6 Software

All the statistical analyses were performed via Stata software (version 17.0). Statistical significance was defined as a two-tailed *p*-value < 0.05.

## 3 Results

### 3.1 Participants

The government staff invited 1,038 potentially eligible older men (aged ≥50 years, local residents, without cognitive impairment or severe illness) to participate. HIV testing identified three participants as HIV positive, who were subsequently excluded. Participants with confirmed positive results were promptly referred to local CDC-designated HIV treatment facilities for counseling, care, and antiretroviral therapy initiation. The remaining 1,035 HIV-negative individuals provided written informed consent and completed the interview process. Six participants discontinued their interviews prematurely because of fatigue and were excluded from the dataset. Consequently, 1,029 participants completed the interviews fully. For the primary analysis examining the relationship between HIV knowledge and stigma, inclusion required participants to report having heard of HIV/AIDS. A total of 841 participants met these criteria, forming the final analytical sample. All subsequent analyses incorporated survey weights to account for the complex sampling design.

### 3.2 Descriptive statistics

The sociodemographic and HIV-related characteristics of the 841 participants are presented in Table 1, showing unweighted counts and weighted percentages. The weighted distribution shows that most participants were from Yibin (63.40%), aged 50–59 years (41.16%), of Han ethnicity (98.19%), and held rural household registration (90.57%). Socioeconomically, 45.26% had only a primary school education, 81.02% worked in basic production occupations, and 46.40% reported monthly incomes of less than 1,000 CNY. Most were married (82.79%). Concerning HIV-related characteristics, over half (57.23%) accessed only zero or one type of HIV/AIDS health education channel, and a small minority (5.47%) reported prior HIV testing.

As summarized in Table 2, the weighted mean HIV knowledge score was 4.39 (SD = 2.13, range 0–8), and the weighted mean HIV stigma score was 31.78 (SD = 5.60, range 10–50). The mean social support score was 7.79 (SD = 1.92, range 2–10), whereas the average sexual attitudes score was 13.19 (SD = 3.59, range 4–20). The standardized composite SES score had a weighted mean of −0.08 (SD = 0.95).

**Table 2:** Weighted descriptive statistics and bivariate associations of continuous variables with HIV stigma.

Variable	*M* (SD)	Range	Association with stigma (*β*)	SE
HIV stigma	31.78 (5.60)	10–50	–	
HIV knowledge	4.39 (2.13)	0–8	−0.307**	0.07
Social support	7.79 (1.92)	2–10	−0.164	0.08
Sexual attitude	13.19 (3.59)	4–20	−0.181***	0.02
Socioeconomic status	−0.08 (0.95)	–	−0.962**	0.17

M = weighted mean; SD = weighted standard deviation. Associations (β) and standard errors (SE) were derived from survey-weighted bivariate linear regression models. *p < 0.05, **p < 0.01, ***p < 0.001.

### 3.3 Bivariate analyses

Bivariate associations between continuous variables and HIV stigma, derived from survey-weighted linear regression models, are presented in Table 2. Higher HIV knowledge, more liberal sexual attitudes, and higher SES each showed significant negative associations with HIV stigma. Social support was not significantly associated with stigma. The results of survey-weighted tests for group differences in HIV stigma levels across categorical variables are shown in Table 3. Significant differences in stigma were observed based on city of residence, age, household registration type, education level, occupation, residential status, number of HIV/AIDS health education channels accessed, and self-reported history of HIV testing. *Post hoc* analyses, accounting for the survey design, clarified specific group contrasts: participants from Liangshan reported significantly lower stigma than those from Chengdu or Yibin; participants aged 60–69 had significantly higher stigma than those aged 50–59; higher educational attainment was associated with significantly lower stigma; professional/technical/managerial workers reported significantly lower stigma than those in basic production occupations; and access to a greater number of health education channels (≥4) was associated with significantly lower stigma. However, no significant differences in stigma emerged across ethnic groups, monthly income categories, or marital status.

**Table 3:** Survey-weighted group differences in HIV stigma scores by participant characteristics.

Variable	*M* (SD)	*F*
City		23.68**
Chengdu	31.46 (6.92)^a^	
Yibin	32.29 (4.38)^a^	
Liangshan	28.13 (8.74)^b^	
Age (years)		6.26*
50–59	30.96 (6.22)^a^	
60–69	32.55 (5.55)^b^	
≥70	32.13 (4.57)^ab^	
Ethnic groups		5.22
Han	31.83 (5.53)	
Ethnic minority	29.49 (8.80)	
Household registration		17.16**
Rural	32.06 (5.41)	
Non-rural	29.12 (6.78)	
Education		27.57**
No education	32.45 (4.41)^a^	
Primary school	32.66 (5.33)^a^	
Junior high school	31.14 (5.83)^b^	
High school and above	29.05 (6.25)^c^	
HIV/AIDS health education (Channel types)		26.78**
0–1	32.68 (4.89)^a^	
2–3	31.16 (5.86)^b^	
≥4	28.35 (7.58)^c^	
Monthly income (CNY)		6.70
No income	31.47 (4.89)	
<1,000	32.67 (4.84)	
1,000–1999	30.97 (6.22)	
2000–2,999	30.95 (6.36)	
3,000–3,999	30.13 (6.28)	
≥4,000	31.61 (6.97)	
Occupation		16.88**
Unemployed or out of work	30.90 (6.22)^ab^	
Basic production	32.17 (5.27)^a^	
Service occupations	30.34 (7.22)^ab^	
Professional, technical, or managerial	28.39 (6.66)^b^	
Marital status		4.93
Married	31.59 (5.64)	
Unmarried	32.88 (4.91)	
Divorced or widowed	32.66 (5.40)	
Residential status		7.19*
Alone	32.66 (5.38)^a^	
With spouse or family only	32.01 (5.44)^ab^	
With spouse and family	30.70 (5.90)^b^	
Self-reported HIV testing		10.87*
Not tested	31.94 (5.47)	
Tested	29.13 (7.10)	

M = weighted mean; SD = weighted standard deviation. Group differences were tested using survey-weighted linear regression models. *F*-statistics and *p*-values account for the complex sampling design. Within each variable, groups with different superscript letters are significantly different at *p* < 0.05 based on *post hoc* pairwise comparisons using survey-weighted linear regression. *p < 0.05, **p < 0.01, ***p < 0.001.

### 3.4 Multivariate analyses

Hierarchical survey-weighted linear regression models were used to examine the associations between HIV knowledge and stigma, adjusting for sociodemographic, HIV-related, and sociocultural covariates (Table 4). Model 1, which contained only control variables, confirmed several factors associated with stigma. Specifically, holding non-rural household registration (*β* = −2.31, *p* < 0.001), accessing more HIV/AIDS health education channels (2–3 types: *β* = −0.97, *p* < 0.01; ≥4 types: *β* = −2.66, *p* < 0.01), and endorsing more liberal sexual attitudes (*β* = −0.20, *p* < 0.001) were each independently associated with lower levels of stigma.

**Table 4:** Hierarchical survey-weighted regression models for the non-linear association of HIV knowledge with stigma.

Variable	Categories	Model 1		Model 2		Model 3	
		*β*	SE	*β*	SE	*β*	SE
City (Ref Chengdu)	Yibin	0.28	0.81	0.29	0.81	0.03	0.80
	Liangshan	−1.55	0.81	−1.56	0.79	−1.08	0.88
Age (Ref 50–59 years)	60–69	0.89	0.46	0.90	0.49	0.93	0.50
	≥70	0.06	0.57	0.08	0.61	0.15	0.59
Ethnic groups (Ref Han)	Ethnic minority	0.82	0.72	0.84	0.70	0.76	1.05
Household registration (Ref Rural)	Non-rural	−2.31***	0.39	−2.31***	0.38	−2.39***	0.33
Marital status (Ref Married)	Unmarried	−0.04	0.63	−0.04	0.64	0.22	0.63
	Divorced or widowed	0.32	0.50	0.32	0.50	0.24	0.44
Residential status (Ref Alone)	With spouse or family only	0.09	0.24	0.09	0.25	0.04	0.25
	With spouse and family	−0.88	0.56	−0.88	0.55	−0.77	0.52
HIV/AIDS health education (Ref 0–1 types)	2–3	−0.97**	0.22	−0.99*	0.29	−1.07**	0.27
	≥4	−2.66**	0.68	−2.68*	0.80	−2.29*	0.73
Self-reported HIV testing (Ref Not tested)	Tested	−1.15	0.85	−1.17	0.88	−1.07	0.93
Social support^a^		0.00	0.09	0.00	0.07	0.01	0.08
Sexual attitude^a^		−0.20***	0.03	−0.20***	0.03	−0.19***	0.03
Socioeconomic status^a^		−0.36	0.17	−0.36	0.18	−0.33	0.18
HIV Knowledge^a^				0.02	0.11	1.71***	0.24
HIV Knowledge × HIV Knowledge^b^						−0.21***	0.02
Constant		34.94***	1.18	34.88***	1.19	32.29***	1.16
*R* ^2^		0.11		0.11		0.13	

All models were estimated using survey-weighted linear regression to account for the complex sampling design. *p < 0.05, **p < 0.01, ***p < 0.001. ^a^Continuous variable. ^b^Quadratic term.

Model 2 introduced HIV knowledge as a linear predictor. The associations for non-rural household registration (*β* = −2.31, *p* < 0.001), access to health education channels (2–3 types: *β* = −0.99, *p* < 0.05; ≥4 types: *β* = −2.68, *p* < 0.05), and liberal sexual attitudes (*β* = −0.20, *p* < 0.001) persisted. HIV knowledge itself showed no significant linear association with stigma (*β* = 0.02, *p* > 0.05). The model’s explanatory power (*R*^2^ = 0.11) was identical to that of Model 1.

**Figure 1:**
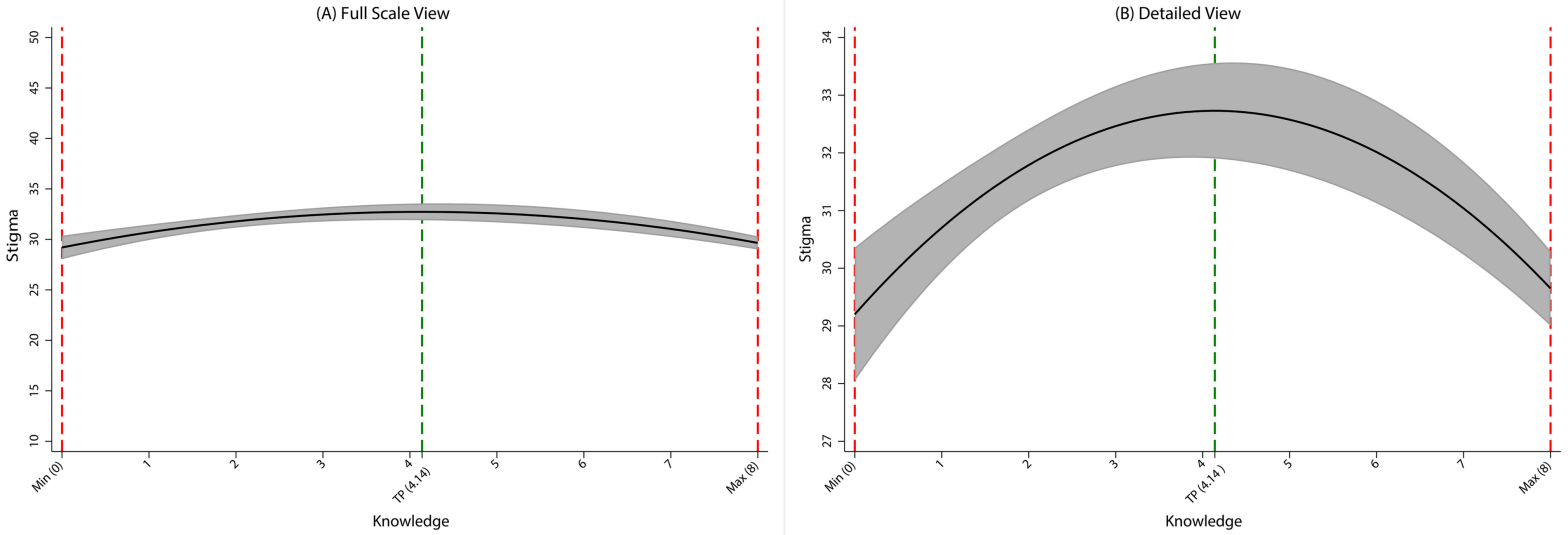
The inverted U-shaped association between HIV knowledge and stigma from the survey-weighted quadratic regression model. **(A)** The association plotted across the full theoretical range of the stigma scale (10–50). **(B)** A detailed view of the association within the range of stigma scores observed in the study sample. The solid line represents the predicted stigma scores, and the shaded area indicates the 95% confidence interval.

**Figure 2:**
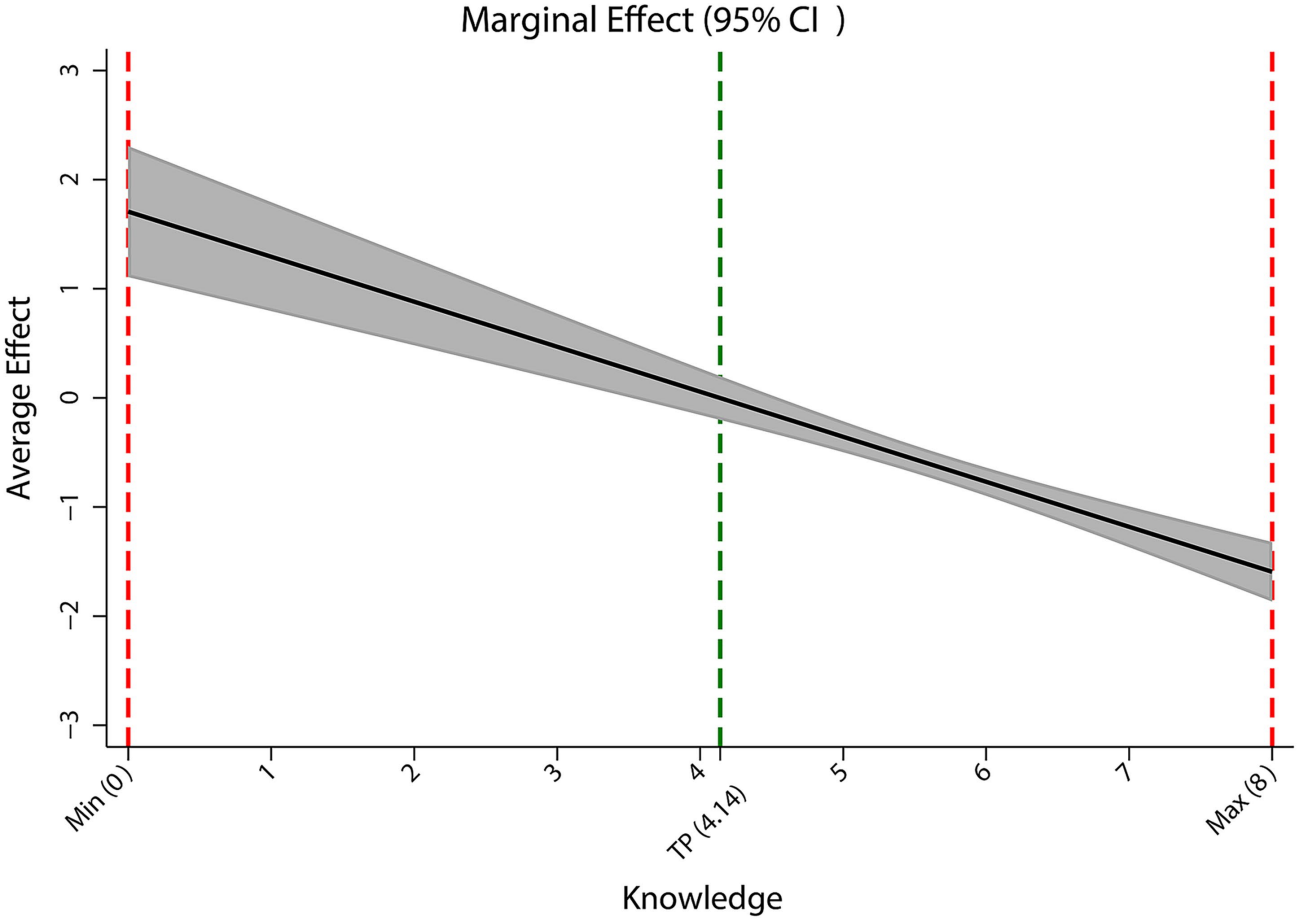
Marginal effect of HIV knowledge on stigma from the survey-weighted quadratic regression model.

Model 3 formally tested for a nonlinear relationship by adding a quadratic term for HIV knowledge (HIV knowledge × HIV knowledge). This revealed a significant inverted U-shaped association: both the linear (*β* = 1.71, *p* < 0.001) and quadratic (*β* = −0.21, *p* < 0.001) terms were highly significant. The inverted U shape was statistically confirmed via the utest command (*t* = 6.96, *p* < 0.001), with the peak stigma occurring at a knowledge score of 4.14 (within the observed 0–8 range). As illustrated in Figure 1A (showing the full scale of the stigma score) and Figure 1B (providing a detailed view of the observed data range), stigma levels initially increased with increasing knowledge, peaked near this turning point, and subsequently declined at higher levels of knowledge. This nonlinear specification enhanced the model fit (*R*^2^ = 0.13). Figure 2 further depicts the marginal effect, showing a positive association between HIV knowledge and stigma below the turning point (with a decelerating trend), crossing zero near the threshold, and transforming into a negative association that accelerated at higher knowledge levels, visually confirming the inverted U shape.

Key covariates demonstrated consistent associations across all models. Non-rural household registration (*β* = −2.39, *p* < 0.001) and liberal sexual attitudes (*β* = −0.19, *p* < 0.001) remained strongly associated with lower stigma in the final model (Model 3). Access to health education channels was also significantly correlated with reduced stigma (2–3 types: *β* = −1.07, *p* < 0.01; ≥4 types: *β* = −2.29, *p* < 0.05). The other covariates included in Model 1 (city of residence, age, ethnicity, marital status, residential status, self-reported HIV testing history, social support, and SES) were not significantly associated with stigma in any of the models.

### 3.5 Robustness checks

Survey-weighted segmented regression and interaction analyses were conducted to validate the stability of the observed inverted U-shaped relationship (Table 5). The sample was stratified at the turning point (knowledge score = 4.14) into lower-knowledge and higher-knowledge groups. In the lower-knowledge group (Model 4), HIV knowledge showed a significant positive association with stigma (*β* = 0.38, *p* < 0.05), corresponding to the ascending segment of the curve. Conversely, in the higher-knowledge group (Model 5), a significant negative linear association was observed (*β* = −0.70, *p* < 0.01), aligning with the descending segment. To formally test the difference in these associations, an interaction term (HIV knowledge group × HIV knowledge) was added to a full-sample model (Model 6). The significant negative coefficient for this interaction (*β* = −1.47, *p* < 0.001) confirms that the relationship between knowledge and stigma differs significantly between the lower- and higher-knowledge groups, thereby robustly supporting the inverted U-shaped pattern.

**Table 5:** Survey-weighted segmented regression and interaction models examining the inverted U-shaped association of HIV knowledge with stigma.

Variable	Categories	Model 4		Model 5		Model 6	
		*β*	SE	*β*	SE	*β*	SE
City (Ref Chengdu)	Yibin	−0.30	0.62	0.36	1.04	0.05	0.81
	Liangshan	−6.34*	1.99	−0.37	1.01	−1.20	0.87
Age (Ref 50–59 years)	60–69	0.53	1.02	0.76	0.34	0.96	0.51
	≥70	−1.12	0.98	1.45*	0.43	0.17	0.62
Ethnic groups (Ref Han)	Ethnic minority	5.35*	1.78	0.25	0.70	0.78	0.97
Household registration (Ref Rural)	Non-rural	−0.95	0.52	−3.38***	0.64	−2.37***	0.32
Marital status (Ref Married)	Unmarried	−1.47*	0.42	1.50	1.16	0.19	0.64
	Divorced or widowed	−0.21	0.48	0.55	0.83	0.23	0.45
Residential status (Ref Alone)	With spouse or family only	−0.39	0.30	0.30	0.53	0.06	0.26
	With spouse and family	−2.03**	0.50	−0.28	0.73	−0.74	0.54
HIV/AIDS health education (Ref 0–1 types)	2–3	0.41	0.21	−1.77*	0.51	−1.05**	0.26
	≥4	−3.16*	1.24	−2.44	1.16	−2.36*	0.76
Self-reported HIV testing (Ref Not tested)	Tested	−1.71*	0.58	−1.47	1.07	−1.08	0.92
Social support^a^		0.13	0.08	−0.26*	0.07	0.00	0.08
Sexual attitude^a^		−0.10**	0.02	−0.29***	0.05	−0.19***	0.03
Socioeconomic status^a^		−0.46*	0.15	−0.19	0.27	−0.33	0.18
HIV Knowledge^a^		0.38*	0.13	−0.70**	0.12	0.61**	0.11
HIV Knowledge group (Ref LTP)	RTP					6.72***	0.77
HIV Knowledge group × HIV Knowledge^b^						−1.47***	0.13
Constant		33.09***	1.59	42.33***	1.51	33.44***	1.22
*R* ^2^		0.11		0.18		0.13	

All models were estimated using survey-weighted linear regression to account for the complex sampling design. LTP = Lower-knowledge group (knowledge score ≤ 4.14); RTP = Higher-knowledge group (knowledge score > 4.14) *p < 0.05, **p < 0.01, ***p < 0.001. ^a^Continuous variable. ^b^Interaction term.

## 4 Discussion

To our knowledge, this cross-sectional study is the first to identify a nonlinear, inverted U-shaped association between HIV knowledge and stigma among older men in Southwest China. Specifically, initial increases in HIV knowledge were linked to increased stigma levels. However, after surpassing a threshold (knowledge score of 4.14 out of 8), further gains in knowledge corresponded with reduced stigma. This inverted U-shaped pattern persisted after adjusting for sociodemographic, HIV-related, and sociocultural variables and was confirmed through segmented regression anchored at the identified peak. These findings challenge the assumption of a consistently inverse linear relationship and provide a novel framework for interpreting inconsistent prior results.

This nonlinear pattern suggests cognitive and contextual influences. At lower knowledge levels, initial exposure to HIV information may amplify preexisting fears or negative stereotypes rather than foster accurate understanding. This is particularly relevant in settings influenced by historical fear-based HIV/AIDS messaging and traditional sexual taboos (4, 5, 30). Fragmented knowledge from limited sources may overemphasize disease severity while neglecting information about prevention, treatment, or the lived experiences of PLWH. This initial phase aligns with fear appeal theory. When individuals lack sufficient knowledge to assess risk rationally, alarming health information may trigger resistance (38). Older men with limited understanding might interpret such information as validating negative attitudes toward PLWH (39), a response potentially intensified by China’s early fear-focused campaigns (30). Partial knowledge may also activate cultural associations linking HIV/AIDS with moral transgression, temporarily heightening stigmatizing attitudes as individuals reconcile new information with existing norms. Within China’s sociocultural context, HIV stigma carries significant moral weight (23), often rooted in perceptions of infection as a consequence of immoral behavior (40). Such associations can threaten social standing (face or “mianzi”), raising concerns about exclusion for PLWH and their families due to perceived moral contamination (18, 41). This moralization of HIV is not unique to China. For instance, in the United States, individuals who strongly associate HIV with immorality are significantly more likely to reject social proximity to PLWH, even after controlling for other factors such as attitudes toward homosexuality (42).

Beyond a critical knowledge threshold, further knowledge acquisition appears to be associated with a more balanced understanding and potentially reduced stigma. Higher knowledge levels may weaken blame attribution toward PLWH, foster empathy, decrease perceived threat, and reduce desired social distance (38, 43, 44). Nevertheless, substantial sociocultural barriers may persist despite knowledge gains. A comprehensive understanding could prove insufficient to overcome the stigma anchored in deeply embedded sociocultural constructs (45). Prevailing moral judgments linking HIV to personal failure, collective beliefs connecting disease to “face” preservation, and community exclusion norms often endure independently of factual knowledge. These forces create structural barriers that are unlikely to be resolved through educational interventions alone (46).

The observed inverted U-shaped relationship between HIV knowledge and stigma offers a nuanced perspective that both contrasts with and integrates findings from prior literature. Globally, studies commonly report an inverse linear association, where greater HIV knowledge is correlated with reduced stigma. For example, research in Vietnam and China has documented fewer stigmatizing attitudes among individuals with higher knowledge levels (19, 20). Similarly, a multicountry analysis across 64 low- and middle-income regions reaffirmed that increased knowledge predicts lower stigma (22), aligning with conventional health promotion frameworks emphasizing knowledge as central to stigma reduction.

However, our findings more closely resonate with studies challenging linear assumptions. In specific contexts, initial knowledge acquisition may heighten stigma. Research in conflict-affected northern Kosovo identified a positive association at lower knowledge levels, which is consistent with the ascending phase of our curve and transitions to a negative association at higher levels, mirroring our threshold effect (26). This parallel suggests that nonlinear dynamics may emerge where sociocultural norms mediate knowledge interpretation.

Notably, our model reconciles inconsistencies in studies reporting weak or non-significant knowledge–stigma links. In Malaysia, high HIV knowledge (mean score at the 70th percentile) has a limited influence on stigma, implying that sociocultural factors outweigh knowledge effects (24). In Nigeria, comprehensive HIV knowledge among women of childbearing age (97.1% of whom lacked such knowledge) did not correlate with reduced stigmatizing attitudes (25). The inverted U-shaped framework contextualizes these null findings: study samples may occupy different curve phases (e.g., prepeak incline or postpeak decline), diluting aggregate linear associations. This highlights the limitations of linear models in capturing threshold dynamics.

Additionally, three factors consistently demonstrated an association with lower stigma levels across all the models: possessing non-rural household registration, accessing HIV information through a greater number of health education channels, and holding more liberal sexual attitudes. These attributes likely reflect greater access to diverse information sources and increased sexual inclusivity. Consequently, individuals with these characteristics may be less inclined to view HIV/AIDS exclusively as a health threat or link it solely to moral transgression, potentially contributing to their reduced stigma (41, 47). These findings also highlight the multidimensional nature of stigma determinants within this population.

Conversely, our analysis revealed no significant associations between stigma levels and social support or SES. This contrasts with prior research. For example, one study of older Chinese adults reported an inverse relationship between social support and stigma toward PLWH (48). This difference may arise from our abbreviated measure of social support, which might not capture its full complexity. Similarly, while research in Ethiopia has linked higher SES to more positive attitudes toward PLWH (49), we observed no such link. The limited variability in rural household registration status, income levels, and occupational categories within our sample may have restricted the range of SES observed, potentially explaining this discrepancy.

These findings suggest that public health interventions targeting HIV stigma among older men in similar contexts may need to be reconceptualized. The observed inverted U-shaped association indicates that the relationship between HIV knowledge and stigma is not uniform but varies by knowledge level. This pattern offers a practical implication for intervention design: strategies could be differentiated based on an individual’s or group’s position on the knowledge spectrum. The HIV knowledge scale employed here, which is brief and used in China’s national surveillance, provides a feasible tool to identify these segments. For those with lower knowledge levels, initial educational efforts might prioritize clarifying modes of transmission and addressing fear-based misconceptions to mitigate potential reinforcement of stigma. For individuals with knowledge levels already above this threshold, interventions could focus on delivering more comprehensive information, integrating content that fosters empathy (such as narratives of PLWH), and challenging sociocultural norms that link HIV to moral failure. This approach moves beyond undifferentiated knowledge dissemination toward more precise strategies that align with the specific relationship between knowledge and stigma observed in this population.

Several limitations should be considered when these findings are interpreted. First, the cross-sectional design precludes causal inferences about the observed relationship between HIV knowledge and stigma. Second, reliance on self-reported data may introduce social desirability bias, potentially underestimating stigma levels. Third, sampling was restricted to three high-HIV-burden areas in Sichuan Province, limiting generalizability to regions with distinct sociocultural or epidemiological profiles. Furthermore, the exclusively male sample precludes generalizing these findings to older women, whose knowledge-stigma relationship may differ due to gendered norms. Fourth, recruitment through primary healthcare institutions might underrepresent older men with limited healthcare access or medical service distrust, potentially introducing selection bias. Finally, unmeasured factors (e.g., religious beliefs) could influence stigma outcomes despite adjustment for key covariates. Longitudinal studies are needed to clarify this relationship’s temporal dynamics and potential causal direction. Qualitative investigations should explore the cognitive processes underlying HIV information interpretation at different knowledge stages. Further research should also examine whether this nonlinear pattern can be generalized to other populations experiencing high stigma and low health literacy across diverse regions where traditional norms intersect with fragmented health information access.

## 5 Conclusion

This cross-sectional study identified a nonlinear, inverted U-shaped association between HIV knowledge and stigma among older men in Sichuan, China. Initial increases in knowledge correlate with heightened stigma, suggesting that sociocultural interpretations of fragmented information may exacerbate negative attitudes in this population. Only beyond a specific threshold does further knowledge acquisition correspond with reduced stigma. These findings challenge conventional linear assumptions about knowledge-stigma relationships and underscore the necessity for contextually tailored interventions. Future stigma-reduction strategies should develop staged interventions tailored to pre-existing knowledge levels, addressing the differential effects revealed in this study while concurrently transforming underlying sociocultural norms.

## Data Availability

The raw data supporting the conclusions of this article will be made available by the authors, without undue reservation.
