# Supplementary material for: The knowledge paradox: an inverted U-shaped association between HIV knowledge and stigma among older men in Sichuan Province, Southwest China
Source: Front Public Health. 2025 Dec 3;13:1685602. doi: 10.3389/fpubh.2025.1685602 (PMC12708549; doi:10.3389/fpubh.2025.1685602)
Supplement: Supplementary file 1 [file Table_1.DOCX]

Supplementary Material 1

The measurement instruments employed in this study for the independent variables, dependent variables, and certain covariates are as follows, demonstrating their specific items.

**HIV Knowledge**

| **Items** | **Yes** | **No** | **Don’t know** |
| --- | --- | --- | --- |
| (1) People living with HIV can be identified by observing their physical appearance. |  |  |  |
| (2) Mosquito bites can spread HIV/AIDS. |  |  |  |
| (3) Eating with people living with HIV can lead to HIV/AIDS. |  |  |  |
| (4) Transfusion of HIV-infected blood can lead to HIV/AIDS. |  |  |  |
| (5) Sharing syringes with people living with HIV can lead to HIV/AIDS. |  |  |  |
| (6) Children born to women living with HIV can acquire HIV/AIDS. |  |  |  |
| (7) Consistent condom use can reduce the risk of HIV/AIDS transmission. |  |  |  |
| (8) Sex with only one partner can reduce the spread of HIV/AIDS. |  |  |  |

**HIV Stigma**

| **Items** | **Strongly Agree** | **Agree** | **Neutral** | **Disagree** | **Strongly Disagree** |
| --- | --- | --- | --- | --- | --- |
| (1) I fear I could become infected with HIV if I were to be exposed to the saliva of a person living with HIV. * |  |  |  |  |  |
| (2) People living with HIV are promiscuous. * |  |  |  |  |  |
| (3) People living with HIV in this community should be treated the same by healthcare professionals as people with other illnesses. |  |  |  |  |  |
| (4) A person living with HIV should be allowed to work with other people. |  |  |  |  |  |
| (5) People living with HIV should be allowed to participate in social events in this community. |  |  |  |  |  |
| (6) People living with HIV should be isolated from other people. * |  |  |  |  |  |
| (7) If a teacher living with HIV is not sick, they should be allowed to continue teaching in school. |  |  |  |  |  |
| (8) People living with HIV in this community face neglect from their families. * |  |  |  |  |  |
| (9) People living with HIV in this community face ejection from their homes by their families. * |  |  |  |  |  |
| (10) People living with HIV in this community are abandoned by their spouse or partner. * |  |  |  |  |  |

Note: * for items with reverse scoring

**Sexual Attitudes**

| **Items** | **Strongly Disagree** | **Disagree** | **Neutral** | **Agree** | **Strongly Agree** |
| --- | --- | --- | --- | --- | --- |
| (1) Nonmarital sexual behavior allows me to indulge myself and seek excitement. |  |  |  |  |  |
| (2) Nonmarital sexual behavior can satisfy my psychological needs. |  |  |  |  |  |
| (3) Men envy men who have multiple sex partners. |  |  |  |  |  |
| (4) Nonmarital sexual behavior can satisfy my physical needs. |  |  |  |  |  |

**Social Support**

| **Items** | **Strongly Disagree** | **Disagree** | **Neutral** | **Agree** | **Strongly Agree** |
| --- | --- | --- | --- | --- | --- |
| (1) If I am in financial need, my family/friends will provide me with economic/material support. |  |  |  |  |  |
| (2) I can get emotional help and support from my family/friends when needed. |  |  |  |  |  |
